# Pulmonary Hypertension in Patients with Idiopathic Pulmonary Fibrosis – The Predictive Value of Exercise Capacity and Gas Exchange Efficiency

**DOI:** 10.1371/journal.pone.0065643

**Published:** 2013-06-20

**Authors:** Sven Gläser, Anne Obst, Beate Koch, Beate Henkel, Anita Grieger, Stephan B. Felix, Michael Halank, Leonhard Bruch, Tom Bollmann, Christian Warnke, Christoph Schäper, Ralf Ewert

**Affiliations:** 1 Department of Internal Medicine B - Cardiology, Intensive Care, Pulmonary Medicine and Infectious Diseases, University of Greifswald, Greifswald, Germany; 2 Department of Internal Medicine I, University Hospital Carl Gustav Carus of TU Dresden, Dresden, Germany; 3 Clinic for Internal Medicine, Trauma Hospital Berlin - Association of BG Hospitals, Berlin, Germany; Vanderbilt University Medical Center, United States of America

## Abstract

Exercise capacity and survival of patients with IPF is potentially impaired by pulmonary hypertension. This study aims to investigate diagnostic and prognostic properties of gas exchange during exercise and lung function in IPF patients with or without pulmonary hypertension.

In a multicentre setting, patients with IPF underwent right heart catheterization, cardiopulmonary exercise and lung function testing during their initial evaluation. Mortality follow up was evaluated.

Seventy-three of 135 patients [82 males; median age of 64 (56; 72 years)] with IPF had pulmonary hypertension as assessed by right heart catheterization [median mean pulmonary arterial pressure 34 (27; 43) mmHg]. The presence of pulmonary hypertension was best predicted by gas exchange efficiency for carbon dioxide (cut off ≥152% predicted; area under the curve 0.94) and peak oxygen uptake (≤56% predicted; 0.83), followed by diffusing capacity. Resting lung volumes did not predict pulmonary hypertension. Survival was best predicted by the presence of pulmonary hypertension, followed by peak oxygen uptake [HR 0.96 (0.93; 0.98)].

Pulmonary hypertension in IPF patients is best predicted by gas exchange efficiency during exercise and peak oxygen uptake. In addition to invasively measured pulmonary arterial pressure, oxygen uptake at peak exercise predicts survival in this patient population.

## Introduction

Idiopathic pulmonary fibrosis (IPF) is a devastating interstitial lung disease usually associated with poor survival [1], [2]. Although respiratory failure is the most frequent cause of death in IPF, mortality is also driven by several comorbidities [2]. Amongst others, there has been a growing appreciation of interceding pulmonary hypertension (PH) in IPF as a important comorbidity with prognostic implications [3], [4], [5], [6]. Depending on its severity, PH may tremendously reduce life expectancy in IPF to less than one year [3]. The severity of lung volume restriction in IPF is well quantified by forced vital capacity (FVC). The degree of FVC reduction as well as FVC decline over time reliably predicts survival in patients with mild to moderate IPF [7], but little is known on how lung volume assessment may mirror circulatory limitations in patients with interceding PH as well.

Impaired pulmonary vascular blood flow due to PH results in reduced exercise capacity and disproportional dyspnoea [6], [8], [9], [10]. Peak oxygen uptake (peakVO_2_) assessed by cardiopulmonary exercise testing (CPET) as well as 6 minute walk distance are significantly impacted by PH [6], [8], [11]. If IPF is complicated by PH, impaired exercise capacity is usually associated with gas exchange inefficiency for carbon dioxide at rest and during exercise, potentially explaining exaggerated dyspnoea [8]. Ventilation to carbon dioxide output ratio (VE/VCO_2_) as a measure of gas exchange efficiency has been shown to be impaired depending on the severity of interceding PH – independent of FVC reduction [8], [9]. However, neither diagnostic nor prognostic implications have been reported for exercise related gas exchange measures. Since transthoracic echocardiography as one standard tool to suspect PH reveals impaired diagnostic accuracy in IPF, additional non-invasive measures predicting interceding PH in this patient populations are of potential interest [12].

The objective of this study is to evaluate lung function variables, exercise capacity and gas exchange efficiency in patients with interceding PH in IPF. As a reference population, IPF patients without PH will be investigated. First, the study aims to describe non-invasive predictive measures for the presence of PH. Second, potential prognostic implications of these measures will be assessed.

## Methods

### Ethics statement

All subjects gave written informed consent. The protocol for investigations, data acquisition and analysis was approved by the local Ethics Committee (Ethics committee of the University of Greifswald, BB 87/11).

### Study population

The study population consist of 135 patients (82 male) with definitive IPF. The diagnoses of definitive IPF were in accordance to current guidelines [13]. All analyses base on a prospectively designed database for IPF patients of the University of Greifswald. The local assessment was obtained at tertiary centres for pulmonary hypertension and IPF in Berlin, Dresden and Greifswald, Germany between 2004 and 2011. All subjects underwent transthoracic echocardiography, right heart catheterization, pulmonary function testing (PFT), diffusing capacity for carbon monoxide and CPET. The mortality follow up was terminated in August 2011. For the prognostic assessment, death and lung transplantation were considered as endpoints.

Subjects with evidence of left heart disease [echocardiography and/or pulmonary capillary wedge pressure (PWP) >14 mmHg by right heart catheterization [14]], diseases other than IPF and/or PH resulting in a life expectancy of less than 24 months or inability to perform CPET due to orthopaedic or neurological impairments were excluded.

Pulmonary hypertension was defined as an increase in mean pulmonary artery pressure (mPAP) to ≥25 mmHg assessed by right heart catheterization [14]. Right heart catheterization was done by Swan-Ganz catheter via Jugular vein at rest in a lying position.

### Lung function and Exercise testing

Immediately prior to CPET, each subject underwent PFT including spirometry, body chamber and diffusing capacity evaluation for carbon monoxide (TLCO and KCO as corrected for alveolar surface area) according to current guidelines [15], [16], [17]. Pulmonary function testing was conducted using a body chamber equipped with a pneumotachograph (VIASYS Healthcare, MasterScreen Body/Diff., JAEGER, Hoechberg, Germany) which meets the American Thoracic Society (ATS) criteria [18].

For the purpose of this study, the following PFT variables were investigated: forced expiratory volume in one second (FEV1) in litres (L), FVC in L, FEV1/FVC ratio, inspiratory vital capacity (IVC) in L, total lung capacity (TLC) in L and KCO in mmol/min/kPa/L.

Cardiopulmonary exercise testing was performed with a physician in attendance according to a modified Jones protocol [19] using a calibrated electromagnetically braked cycle ergometer (Ergoselect 100, Ergoline, Germany). Gas exchange and ventilatory variables were analysed breath by breath using a VIASYS HEALTHCARE system (Oxycon Pro, Combitox mask) which had been recalibrated just prior to each test. 12-lead ECG's were recorded during rest and every minute thereafter; pulse oximetry was monitored continuously; and blood pressure was obtained by cuff sphygmomanometer every two minutes. Prior to the test, patients were encouraged to reach maximal exhaustion; during exercise no further motivation was utilised. All tests were performed according to current guidelines for CPET [20], [21].

Values for minute ventilation (VE), tidal volume (Vt), VO_2_, and carbon dioxide output (VCO_2_) were acquired on a breath-by-breath basis and averaged over 20 s intervals. PeakVO_2_ was defined as the highest 20-s average of VO_2_ in the last minute of the exercise test. Ventilatory efficiency, expressed as the relation of VE and VCO_2_, was defined as the slope of the regression of both parameters excluding excess hyperventilation at the end of exercise (VE vs. VCO_2_ slope). The anaerobic threshold (AT) was determined according to Wasserman et al and quantified as oxygen uptake at AT (VO_2_@AT) [22]. The breathing reserve (VE/MVV) was calculated as maximal VE in relation to maximal voluntary ventilation (MVV). MVV was calculated by FEV1×41.

At peak exercise, dynamic flow-volume loops were documented and evaluated for inspiratory capacity (IC). The manoeuvre was assessed for peak Vt in relation to peak exercise IC (Vt/IC) [8].

The following CPET variables were assessed for the purpose of this investigation: peakVO_2_ and VO_2_@AT (mL/min), VE vs. VCO_2_ slope, VE_max_ and Vt_max_ (L),m Vt_max_/IC and VE/MVV.

### Statistical analysis

Besides absolute values relative % predicted values were analysed. The following sets of reference were applied to calculate % predicted values (_pred_): PFT by Koch et al [23], TLC by Hansen [24], KCO by Gläser et al [25], exercise variables by Wasserman et al, Koch et al and Gläser et al [22], [26], [27], [28].

Predicting prognosis based on variables for age, sex, FEV1_pred_, FVC_pred_, KCO_pred_, peakVO_2pred_, VO_2_@AT_pred_ and VE vs. VCO_2_ slope_pred_. In addition, absolute values of all variables were analysed.

Continuous data are expressed as median (25th and 75th quartile). The Mann-Whitney U-test (continuous data) or X-test (nominal data) were used for comparison of IPF patients with PH or without PH. Cox proportional hazards analysis was performed to assess the association between pulmonary variables and the end point. Hazards ratios and 95% confidence intervals for risk factors as well as levels for x^2^-test (likelihood ratio test) are given. Kaplan Meier cumulative survival plots were constructed (SPSS version 20.0). Differences in survival were analyzed by log-rank test. Cut-off values for best discrimination were calculated using receiver-operating-characteristics. A p-value<0.05 was considered significant. Statistical analysis was carried out using SPSS (SPSS, Version 20.0 for Windows; SPSS Inc., Chicago, IL), SAS 9.1 software (SAS Institute Inc., Cary, NC) and R (free shareware, www.r-project.org).

## Results

The study population bases upon 135 subjects (82 males) with definitive IPF. The median age was 64 years (25th percentile: 56; 75th percentile: 72). Of those, 73 patients had PH [mPAP: median, 34 (25^th^ percentile: 27; 75^th^ percentile: 43) mmHg; PWP: median 8 (25^th^: 5; 75^th^: 12) mmHg; cardiac index: median 2.13 (25^th^: 1.83; 75^th^: 2.44) l/min/m^2^)]. Patients with and without PH did not differ concerning sex [men: 64.8 vs. 55.0%, p = 0.271], height [171 (164; 178) vs. 170 (164; 175) cm, p = 0.299] and weight [75 (65; 85) vs. 72 (63; 85) kg, p = 0.466], but in age [63 (52; 68) vs. 68 (58; 74) years, p = 0.026].

Lung function and CPET variables of both groups are given in table 1. With the exception of KCO there were no differences in PFT variables. Patients with PH had lower VO_2_@AT and peak exercise and had significantly higher VE vs. VCO_2_ slope values. Ventilatory constrains (VE, Vt, VT_max_/IC and VE/MVV) did not differ.

**Table 1:** Lung function and gas exchange characteristics of the patient population.

		absolut value			Relative value (%pred)		
		IPF-PH	IPF-non-PH		IPF-PH	IPF-non-PH	
	unit	Median	Median	P	Median	Median	P
		[25th; 75th]	[25th; 75th]		[25th; 75th]	[25th; 75th]	
**Pulmonary Function Testing**							
FEV1	L	1.67	1.78	0.570	55.82	67.02	0.035
		[1.28; 2.27]	[1.34; 2.31]		[45.52; 73.36]	[49.64; 82.34]	
FVC	L	2.11	2.32	0.667	56.22	58.94	0.116
		[1.58; 2.91]	[1.71; 2.93]		[42.88; 66.51]	[46.24; 76.68]	
FEV1/FVC	ratio	0.84	0.82	0.765	101.28	100.36	0.788
		[0.74; 0.90]	[0.75; 0.89]		[88.71; 108.97]	[89.96; 107.47]	
IVC	L	2.21	2.31	0.820	61.20	59.04	0.355
		[1.48; 3.10]	[1.74; 2.91]		[44.00; 69.45]	[47.49; 77.46]	
TLC	L	4.43	4.06	0.380	70.57	69.07	0.900
		[3.29; 5.57]	[3.33; 4.87]		[57.05; 81.88]	[58.10; 82.52]	
KCO	mmol/min/kPa/L	0.54	0.89	<0.001	38.97	67.80	<0.001
		[0.37; 0.76]	[0.64; 1.16]		[25.89; 56.68]	[47.76; 83.48]	
**Cardiopulmonary Exercise Testing**							
peakVO_2_	mL/min	810.4	1161.3	<0.001	43.21	68.70	<0.001
		[579.6; 1012.0]	[870.1; 1493.1]		[33.86; 53.24]	[49.08; 78.71]	
VO_2_@AT	mL/min	631.5	866.78	0.005	57.15	78.04	<0.001
		[482.7; 765.4]	[598.41; 989.00]		[44.05; 67.24]	[54.04; 95.12]	
VE vs. VCO_2_ slope	ratio	48	32	<0.001	189.42	126.72	<0.001
		[40; 64]	[30; 37]		[157.25; 243.00]	[108.84; 146.24]	
VE_max_	L	47.45	43.46	0.479	78.66	73.60	0.994
		[35.50; 62.25]	[36.00; 60.00]		[58.71; 95.16]	[61.69; 90.19]	
Vt_max_	L	1.11	1.30	0.433	53.56	61.75	0.056
		[0.84; 1.60]	[1.00; 1.54]		[40.14; 63.75]	[49.65; 71.24]	
Vt_max_/IC	ratio	0.83	0.75	0.669	137.5	124.83	0.669
		[0.60; 1.00]	[0.65; 0.89]		[100.0; 166.7]	[108.33;147.83]	
VE/MVV	ratio	0.6	0.60	0.827	83.33	83.47	0.823
		[0.5; 0.8]	[0.52; 0.72]		[69.44; 111.11]	[72.50; 100.56]	

Comparison of IPF patients with and without PH. Results (median and 25^th^; 75^th^ percentile) are given as absolute and % predicted values.

### PH prediction

Potentials of prediction of interceding PH as well as statistical significant cut offs are given in table 2. Highest AUC was seen for VE vs. VCO_2_ slope_pred_, followed by peakVO_2pred_ and KCO_pred_. Applying %_pred_ values showed statistical significant superiority to absolute values (data not shown). AUC for KCO_pred_ was 0.751 (cut off 57.4), for peakVO_2 pred_ 0.832 (cut off 56.3), for VO_2_@AT_pred_ 0.743 (cut off 71.9) and for VE vs. VCO_2_ slope_pred_ 0.938 (cut off 152.4). Combined endpoints consisting of each of the significant variables did neither improve AUC nor sensitivities/specificities (data not shown).

**Table 2:** Predictive potencies of lung function and exercise variables for the presence of PH.

Variable	AUC [95%-CI]	Cut off	SN [%]	SP [%]	PPV [%]	NPV [%]
**Pulmonary Function Testing**						
KCO_pred_	0.751 [0.654;0.847]	≤57.4	78.0	67.3	69.6	76.1
FEV1_pred_	0.609 [0.510;0.709]	≤62.0	59.7	60.3	63.5	56.5
**Cardiopulmonary Exercise Testing**						
VE vs. VCO_2_slope_pred_	0.938 [0.892;0.984]	≥152.4	87.2	88.4	89.1	86.4
peakVO_2pred_	0.832 [0.753;0.911]	≤56.3	83.0	68.9	75.9	77.4
VO_2_AT_pred_	0.743 [0.631;0.854]	≤71.9	88.9	56.4	65.3	84.6

Area under the curve (AUC) values and their confidence interval (95%-CI), sensitivity (SN), specificity (SP), positive (PPV) and negative predictive values (NPV) are given. Diagnostic cut offs in % predicted.

### Prognosis in IPF

**Figure 1:**
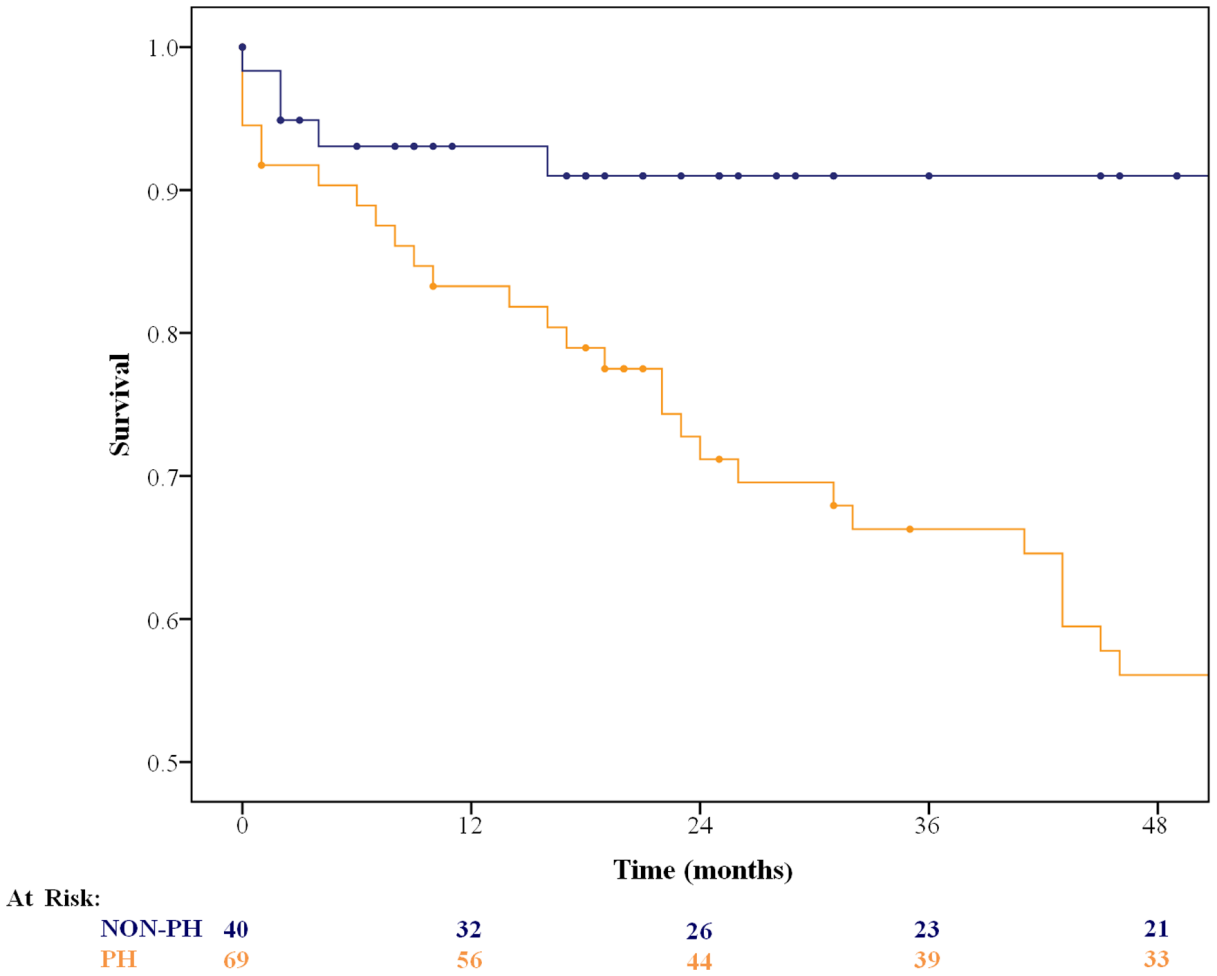
Survival of 133 patients with IPF with and without interceding pulmonary hypertension. Non-PH (blue line), PH (orange line).

Survival analysis is shown in figure 1. During the follow up, 37 patients died and 6 underwent lung transplantation. For two subjects, no follow up information were available, reducing the study sample to 133 patients. Overall survival in the IPF population was 87.5% at one year and 79.3% at two years. The statistically most powerful prognosticator was the presence of PH assessed by right heart catheterization [mPAP: hazard ratio 1.07 (1.04; 1.11); ChiSq 17.22; ProbChiSq <0.0001].

In the overall IPF population KCO_pred_, peakVO_2pred_ and VO_2_@AT_pred_ revealed significant prognostic properties (table 3) in the univariate analysis. In the multivariate analysis, besides the presence of PH only peakVO_2pred_ was independently predictive for survival. Again, applying %_pred_ values showed statistically significant superiority to absolute values (data not shown).

**Table 3:** Prognostic evaluation of lung function and gas exchange.

Variable	Hazard ratio	ChiSqare	ProbChiSq	Total	Events
age	1.02 (0.99; 1.05)	2.54	0.111	133	43
sex	0.78 (0.41; 1.47)	0.62	0.432	133	43
FEV1_pred_	0.99 (0.98; 1.01)	0.35	0.553	125	38
FVC_pred_	0.99 (0.97; 1.00)	2.69	0.101	125	38
KCO_pred_	0.98 (0.96; 1.00)	5.84	0.016	102	28
peakVO_2pred_	0.96 (0.93; 0.98)	11.98	0.001	98	28
VO_2_@AT_pred_	0.97 (0.95; 1.00)	5.68	0.017	75	18
VE vs. VCO_2_ slope_pred_	1.00 (1.00; 1.01)	2.63	0.105	90	25

Prognostic relevance of lung function and exercise related parameters assessed by univariate analysis.

Subgroup analyses for subjects with PH again revealed the prognostic properties of the presence of PH, but other significant associations could not be shown.

## Discussion

In terms of the hypothesis framing this study, patients with interceding PH in IPF show significant differences in exercise capacity, gas exchange as quantified by ventilatory efficiency for carbon dioxide and diffusing capacity, independent of the severity of restrictive lung disease. The presence of PH is best predicted by VE vs. VCO_2_ slope and peakVO_2_ in our patients. Survival is severely impaired in PH patients. Besides the presence of PH as invasively assessed by mPAP, prognosis is best predicted by oxygen uptake at peak exercise.

Advanced interstitial lung diseases impair exercise capacity and increase dyspnoea during exercise [8], [9], [29], [30]. In IPF, exercise capacity is further diminished by coexisting PH, reflected in a further impairment of peakVO_2_ during incremental exercise tests as well as in 6 minute walk distance [6], [8]. As demonstrated previously, reduced exercise capacity is accompanied by increasing ventilatory constrains to eliminate carbon dioxide as quantified by ventilatory inefficiency, both independent of the severity of restrictive lung disease due to IPF [8], [9]. During incremental exercise, exercise capacity depends on any organ system involved in the coupling of respiration, gas exchange and cellular metabolism. In patients with advanced IPF, one may expect that the restriction in exercise capacity is due to ventilatory mechanics. In the present study, lung volumes did not differ between PH and non-PH subjects. Furthermore, measures during exercise to quantify ventilatory mechanical constrains such as breathing reserve and Vt/IC relationship turned out to be moderately abnormal and did not separate between patients with and without PH. Therefore, we assume that diminished lung mechanics contribute to impaired exercise capacity and ventilatory inefficiency in the overall population of IPF patients. Our as well as previously published findings imply that interceding PH in IPF results in a further limitation due to circulatory impairment [8], [9].

To the best of our knowledge this is the first study investigating the potencies of PFT and CPET measures in their predictive power to suspect PH in IPF. Diagnosing PH even in IPF implies invasive assessment of mPAP, and in the majority of cases suspicion of PH will base on transthoracic echocardiography [14], [31]. Since transthoracic echocardiography is complicated by impaired sensitivity and specificity in IPF patients, we suggest PFT and CPET measures as useful additional tools in the diagnostic work up [12]. Boutou et al described VE/VCO_2_ ratio at AT as an independent predictor of PH in 81 IPF patients [9]. However, this study bases on echocardiography only and does not provide a usable cut off for VE/VCO_2_ ratio. The present study shows that PH is best predicted by VE vs. VCO_2_ slope_pred_, followed by peakVO_2pred_, KCO_pred_ and VO_2_@AT_pred_ applying relative (%predicted) rather than absolute values and adjusting for potential interfering factors, such as age, sex, height and weight. This theoretical rational is further verified by our analyses, since relative values showed statistical superiority.

This study further contributes to our knowledge on how lung function and exercise capacity will contribute to the prognostic assessment in IPF. Longitudinal decline in FVC has been shown to be a reliable prognosticator in IPF [7]. The data shown by du Bois base on patients evaluated within interventional trials, and it remains unclear whether these trials include substantial numbers of patients with interacting PH [32], [33]. Fell et al outlined peakVO_2_ as prognostically important for patients with different entities of interstitial lung disease [29]. Besides the heterogenic population the proportion of PH patients was not investigated [29]. In our study, patients with PH showed poor survival compared to the overall IPF population. The major impact on survival was due to the presence of PH as assessed by invasive diagnostics. In addition, peakVO_2pred_ showed prognostic properties. Focusing on the PH subgroup, no statistical significant predictors were shown - potentially because of an underpowered subgroup of PH patients. Further studies are needed to clarify prognostication in this specific population.

The strength of the study is the multi-centre design with a standardized study protocol for exercise testing and lung function. In this setting, “real life” IPF patients of centres specialized in IPF and PH have been investigated, the potential biases due to interventional therapeutic trials or waiting lists for lung transplantation have been avoided. All evaluations base on invasively assessed PH. Of course, this study has limitations. First, the study was not powered to investigate further subgroups of IPF patients for prognostication since just 19 patients in the non-PH group reached an endpoint. Second, the majority of patients were investigated in specialized centres for PH and IPF resulting in some recruitment bias. We are unable to quantify this bias. Finally, the data set lacks measurements of arterial blood gases in a substantial number of patients and consequently, these measures could not be analysed. Unfortunately, the same is true for 6 minute walk distance.

## Conclusion

In IPF, coexisting PH further impairs diffusing capacity, exercise capacity and ventilatory efficiency. The clinical and prognostic consequences of PH might not adequately be assessed by resting PFT only. Exercise related measures in addition to PFT will help to evaluate IPF patients, since PH in this patient population has a major prognostic impact. Ventilatory efficiency quantified by VE vs. VCO_2_ slope_pred_ is the most reliable predictor of PH. In the overall IPF population, besides the presence of the PH survival is best predicted by peak oxygen uptake.
